# The Computational Revolution in Natural Product Research: A Data-Driven Roadmap for Next-Generation Drug Development

**DOI:** 10.3390/biology15080632

**Published:** 2026-04-17

**Authors:** Mia Yang Ang, Siew Woh Choo

**Affiliations:** 1 Department of Biomedical Sciences, Sir Jeffrey Cheah Sunway Medical School, Faculty of Medical and Life Sciences, Sunway University, Sunway City, Petaling Jaya 47500, Selangor, Malaysia; 2 Sunway Microbiome Centre, Faculty of Medical and Life Sciences, Sunway University, Sunway City, Petaling Jaya 47500, Selangor, Malaysia; 3 Zhejiang-Malaysia Joint Laboratory for Rare Medicinal Resources, Wenzhou-Kean University, 88 Daxue Road, Ouhai, Wenzhou 325060, China; 4 College of Science, Mathematics and Technology, Wenzhou-Kean University, 88 Daxue Road, Ouhai, Wenzhou 325060, China; 5 International Frontier Interdisciplinary Research Institute (IFIRI), Wenzhou-Kean University, Wenzhou 325060, China; 6 Dorothy and George Hennings College of Science, Mathematics and Technology, Kean University, 1000 Morris Ave, Union, NJ 07083, USA

**Keywords:** natural products, genome mining, sustainable drug development, big data analytics, chemoinformatics

## Abstract

Natural products (NPs) have historically provided the foundational scaffolds for drug development, yet traditional bioprospecting faces critical limitations: high rediscovery rates, laborious isolation workflows, and substantial attrition during clinical translation. The emergence of big data technologies is fundamentally transforming this landscape, enabling a shift from serendipity-based discovery toward systematic, data-driven approaches. This review examines how the integration of artificial intelligence (AI), machine learning (ML), and multi-omics datasets is accelerating natural product research across three key domains: (1) genome mining for biosynthetic gene cluster identification using platforms such as antiSMASH, (2) cheminformatics-driven prediction of structure–activity relationships and ADMET properties, and (3) metabolomics-guided dereplication to prioritize novel bioactive scaffolds. We evaluate the convergence of genomics, metabolomics, and computational chemistry in enabling *in silico* lead optimization and the discovery of cryptic metabolites from previously inaccessible microbial taxa. While challenges in data standardization and scalability persist, the synergy between big data and NP research is accelerating clinical translation. Despite persistent challenges in data standardization, scalability, and equitable benefit-sharing, the convergence of big data and NP research is poised to redefine drug development. These advances position computational NP research as a cornerstone of next-generation drug development.

## Simple Summary

This review explores how the integration of big data and artificial intelligence is revitalizing natural product drug discovery. While nature has historically provided our most successful medicines, traditional discovery methods have stalled due to high costs and the frequent rediscovery of known compounds. By combining genomic “blueprints” with machine learning and advanced chemical analysis, this research highlights a shift toward a “digital renaissance” in bioprospecting. These computational tools enable scientists to rapidly identify novel drug leads and predict their safety, positioning data-driven natural product research as a cornerstone for the next generation of effective and sustainable medicine.

## 1. Introduction

Natural products (NPs) remain humanity’s most productive source of drug leads [1]. The history of public health is punctuated by landmark NP discoveries, most notably the antibiotic penicillin [2] and the potent anticancer agent paclitaxel [3]. These naturally derived scaffolds exhibit a degree of chemical diversity and biological specificity that is often unattainable through traditional synthetic drug libraries [4]. By evolving within complex ecological niches, these molecules are inherently pre-validated to interact with biological macromolecules, offering unique mechanisms of action.

However, despite this immense pharmacological potential, NP discovery has stalled. The process of extracting, isolating, and characterizing bioactive metabolites from complex biological matrices is notoriously time-intensive and cost-prohibitive [5]. Furthermore, the limited natural abundance of specific compounds and their structural intricacies—such as multiple chiral centers and dense functionalization—often impede large-scale exploration and clinical translation [6]. These barriers, combined with the pharmaceutical industry’s shift toward high-throughput synthetic screening in the 1990s, reduced investment in natural product programs for nearly two decades (Table 1) [7].

Three converging technologies are now reversing this decline [8]. First, high-throughput next-generation sequencing (NGS), coupled with sophisticated bioinformatics tools, now allows researchers to mine microbial and plant genomes for biosynthetic gene clusters (BGCs). This enables the discovery of novel metabolites from previously untapped sources, including unculturable microorganisms and extreme environments [9,10]. Second, parallel advancements in metabolomics and cheminformatics have provided deeper insights into structure–activity relationships (SAR) and pharmacokinetic profiles, bridging the gap between chemical complexity and clinical relevance [11,12]. Third, machine learning methods trained on expanding chemical and bioactivity databases can now predict compound function, prioritize leads, and guide biosynthetic engineering [13].

Here we examine how these data-intensive approaches are reshaping NP research. We assess how data-centric approaches are revolutionizing key phases of research, from the in silico identification of bioactive leads to the elucidation of complex biological pathways. Through the analysis of successful case studies and the discussion of persistent challenges, such as data standardization, computational complexity, and ethical frameworks, this paper provides a roadmap for future innovations in the field. By synthesizing these technological advancements, we aim to demonstrate the potential of big data to reinvigorate NP research and drive the next generation of drug development.

**Table 1:** Comparative characteristics of NPs and Synthetic Compound libraries in drug discovery. Key differences in chemical properties, discovery workflows, and translational challenges. Neither approach is universally superior; optimal strategy depends on therapeutic target and program objectives.

Feature	Natural Products (NPs)	Synthetic Compound Libraries	References
Chemical Diversity	Exceptional structural complexity; high density of chiral centers and rare functional groups.	Targeted diversity; often constrained by existing building blocks and “Lipinski’s Rule of Five.”	[4,14]
Biological Specificity	Evolutionarily optimized for high affinity and specificity toward biological targets.	Variable; often requires extensive optimization to minimize off-target interactions.	[4,15]
Mechanisms of Action	Frequently address novel or “undruggable” pathways via unique scaffolds.	Predominantly target well-characterized, established biological pathways.	[1,15]
Discovery Workflow	Traditionally resource-intensive; involves isolation, dereplication, and characterization.	Accelerated via combinatorial chemistry and high-throughput screening (HTS).	[1,5]
Chemical Tractability	Structural complexity often limits semi-synthetic modifications and lead optimization.	Highly amenable to rational drug design and iterative synthetic modifications.	[4,16]
Scalability	Often limited by low natural titer and ecological constraints; requires fermentation or total synthesis.	Highly scalable through standardized industrial manufacturing processes.	[6,17]
Primary Bottlenecks	Isolation kinetics, supply sustainability, and structural elucidation.	Restricted chemical space and potential for lower biological relevance.	[4,18]

## 2. The Importance of Natural Products in Drug Development

NPs occupy a distinct chemical space shaped by evolutionary selection for biological activity [19]. Derived from a diverse array of taxa, including terrestrial plants, fungi, bacteria, and marine invertebrates, these metabolites feature intricate architectures characterized by high stereochemical complexity and architectural rigidity [20]. Unlike synthetic libraries, which are often “flat” and follow narrow design rules, NP scaffolds possess a high density of *sp*^3^-hybridized carbons, multiple chiral centers, and unique functional groups [21,22,23]. This inherent complexity allows NPs to interact with biological macromolecules, such as protein-protein interfaces, with a degree of specificity and affinity that synthetic small molecules rarely achieve [24]. However, this same complexity poses a significant challenge for chemical tractability, as the total synthesis and rational modification of such structures remain resource-intensive and costly [16].

NPs have delivered foundational therapies across major disease areas, particularly in oncology, infectious diseases, and inflammatory disorders [25]. In oncology, paclitaxel and the vinca alkaloids remain frontline agents [26]. In infectious disease, vancomycin [27] and the β-lactams defined antibiotic therapy for decades. Secondary metabolites like curcumin continue to provide promising scaffolds for anti-inflammatory research [28]. More recent derivatives, including the rapamycin analogue everolimus and the halichondrin-derived eribulin, demonstrate that natural scaffolds continue to yield clinically differentiated medicines (Table 2).

Yet conventional discovery pipelines face diminishing efficiency. The reliance on bioassay-guided fractionation often leads to the repeated isolation of known compounds, a process that is both time-consuming and prone to high attrition [5]. Furthermore, challenges regarding metabolic stability, systemic bioavailability, and low natural abundance often hinder the transition from laboratory “hit” to clinical “lead” [6]. These limitations are further exacerbated by ecological concerns regarding the over-harvesting of rare species [29]. These pressures are driving a strategic shift toward genome-informed and computationally guided discovery—approaches that can identify novel chemistry while reducing dependence on destructive extraction [17].

**Table 2:** Landmark natural product-derived drugs across therapeutic areas. Representative examples illustrating the taxonomic diversity of source organisms and the breadth of clinical applications. Drugs are grouped by primary therapeutic indication.

Drug	Natural Source	Therapeutic Application	Key References
Penicillin	*Penicillium chrysogenum* (Mold)	Antibiotic (Bacterial infections)	[30]
Paclitaxel	*Taxus brevifolia* (Pacific yew tree)	Oncology (Ovarian, breast cancer)	[31]
Aspirin	*Salix* species (Willow bark)	Analgesic, Anti-inflammatory	[32]
Vancomycin	*Amycolatopsis orientalis* (Bacteria)	Antibiotic (Gram-positive infections)	[33]
Quinine	*Cinchona* (Tree bark)	Antimalarial	[34]
Lovastatin	*Aspergillus terreus* (Fungi)	Lipid-lowering (Statin)	[35]
Doxorubicin	*Streptomyces peucetius* (Bacteria)	Oncology (Leukemia, Lymphoma)	[36]
Cyclosporine	*Tolypocladium inflatum* (Fungi)	Immunosuppressant	[37]
Artemisinin	*Artemisia annua* (Sweet wormwood)	Antimalarial	[38]
Erythromycin	*Saccharopolyspora erythraea*	Antibiotic (Bacterial infections)	[39]
Curcumin	*Curcuma longa* (Turmeric)	Anti-inflammatory research	[40]
Rifamycin	*Amycolatopsis rifamycinica*	Antibiotic (Tuberculosis)	[41]

## 3. Data-Driven Approaches in Natural Product Research

The scale of available biological and chemical data has transformed NP research [8]. Genomic sequences, metabolomic profiles, bioactivity measurements, and structural annotations can now be integrated within unified computational frameworks, enabling systematic exploration that was previously impractical.

**Figure 1:**
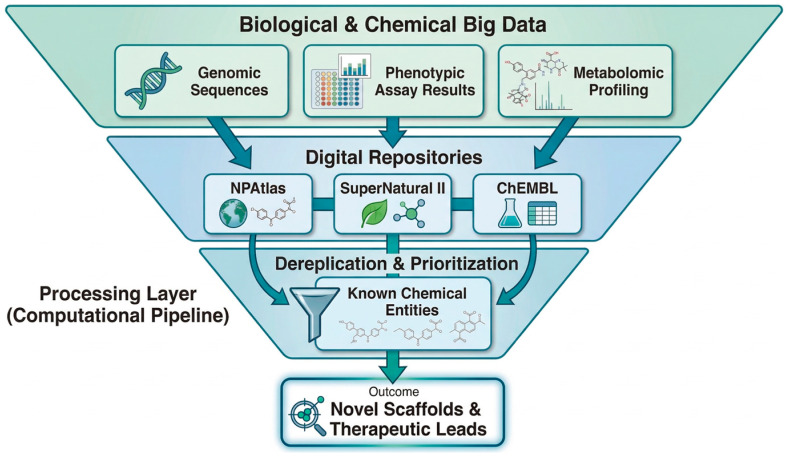
Data-Driven Workflow for NP Drug Discovery. Raw data streams—genomic sequences, metabolomic profiles, and bioactivity measurements—are integrated through curated repositories (NPAtlas, SuperNatural II, and ChEMBL) and processed via computational pipelines that perform dereplication, structural annotation, and bioactivity prediction. This framework enables systematic prioritization of novel scaffolds over known compounds, shifting discovery from phenotypic screening toward genome-guided target identification.

Public databases support this infrastructure. Among of these databases are NPAtlas [42], SuperNatural II [43], and ChEMBL [44]. NPAtlas catalogues microbial natural products with validated structural and taxonomic metadata. SuperNatural II is a comprehensive, publicly accessible database of natural products and their derivatives that are considered “ready for virtual screening.” ChEMBL links compounds to quantitative bioactivity data across target classes. Together, these resources enable rapid dereplication—distinguishing novel chemistry from known compounds early in the discovery process—and support machine learning applications that require large, annotated training sets (Figure 1).

Two complementary strategies drive current discovery efforts. Genome mining identifies biosynthetic gene clusters encoding secondary metabolite pathways, revealing synthetic potential that often exceeds observed chemical output by an order of magnitude [45]. Metabolomics captures the compounds actually produced under defined conditions, providing ground-truth chemical phenotypes [46]. Integrating these approaches—correlating predicted biosynthetic capacity with detected metabolites—enables prioritization of “cryptic” or conditionally expressed pathways and guides efforts to activate silent gene clusters through heterologous expression or culture manipulation.

In this context, prioritization refers to the computational ranking of isolates based on a ‘Novelty Score’—calculated via tools like BiG-SCAPE [47] and a ‘Bioactivity Probability’ derived from ML models. By filtering out clusters or metabolites with high similarity to known entries in the MIBiG [48] or NPAtlas [49] databases, researchers can focus resources on ‘dark matter’ scaffolds that represent truly unique chemical space.

## 4. Computational and Multi-Omics Approaches

The advancement of Artificial Intelligence (AI) and Machine Learning (ML) has established new frontiers in NP research by providing the analytical rigor necessary to interpret high-dimensional datasets [50]. Deep learning architectures predict bioactivity from molecular structure, identify structure–activity relationships across large compound sets, and prioritize leads based on predicted ADMET (Absorption, Distribution, Metabolism, Excretion, and Toxicity) profiles—filtering compounds with poor pharmacokinetic or toxicity liabilities before synthesis [13,27,51,52,53]. These approaches complement physics-based methods: molecular docking estimates binding affinity, while molecular dynamics simulations assess conformational stability and binding kinetics at atomistic resolution. Beyond traditional metabolomics, the integration of lipidomics has emerged as an important tool for the massive investigation of lipid-based metabolites and cell membrane components [54]. Given that many natural products exert their effects by modulating membrane fluidity or signaling lipids, lipidomic profiling provides a systems-level view of how NPs interact with the cellular lipidome, offering insights that are often missed by broader metabolomic screens.

Multi-omics integration extends beyond individual compound discovery to system-level pathway elucidation. While correlating transcriptomic responses with metabolite production is conceptually powerful, it faces significant technical hurdles. Metatranscriptome datasets are often characterized by extreme sparsity and ‘zero-inflation’, where the absence of a signal does not necessarily imply the absence of expression [55]. Moving forward, the field is transitioning toward Zero-Inflated Negative Binomial (ZINB) models [56] and deep-learning-based imputation [57] to distinguish biological silence from technical noise, ensuring that multi-omics correlations remain statistically sound.

## 5. Genome Mining and Biosynthetic Gene Cluster Analysis

Microbial and plant genomes encode far more secondary metabolite pathways than phenotypic screens detection [58]. Genome mining systematically identifies biosynthetic gene clusters, revealing this hidden biosynthetic capacity [59]. Automated annotation platforms, particularly antiSMASH (Antibiotics and Secondary Metabolite Analysis Shell), have become indispensable for identifying and annotating these BGCs, effectively streamlining a process that was previously labor-intensive [60]. Beyond primary cluster detection, these tools facilitate the identification of homologous BGCs across diverse species, providing critical insights into evolutionary conservation and the resulting chemical diversity. This informatics-led strategy has enabled the targeted exploration of underexplored microbial taxa, such as rare actinomycetes and marine bacteria, which represent a significant but previously inaccessible reservoir of bioactive metabolites. Beyond primary detection, antiSMASH [61] facilitates the prediction of chemical scaffolds; however, this capability is currently most robust for modular biosynthetic gene clusters, such as Polyketide Synthases (PKS) and Non-Ribosomal Peptide Synthetases (NRPS). For these classes, scaffold prediction relies on identifying conserved substrate-specification domains (e.g., A-domains) and comparing them to experimentally validated clusters in the MIBiG database [48]. For more divergent or cryptic BGCs, scaffold prediction remains a significant computational challenge [62].

This approach has proven especially valuable in antibiotic discovery [63]. Facing rising multidrug-resistant pathogens, researchers have used genome mining to identify clusters encoding compounds with unprecedented mechanisms of action [64]. For example, systematic analysis of underexplored taxa—rare actinomycetes, marine bacteria, and fungal endophytes—has yielded new antibiotic and antifungal scaffolds targeting previously unexploited pathways [65]. Furthermore, genome mining has expanded the known scope of chemical diversity in natural products by enabling the systematic prediction of novel structural frameworks [66]. By studying variations in BGC organization across species, scientists can uncover unique biosynthetic pathways that lead to compounds with diverse pharmacological activities. This approach not only enhances the discovery of new drugs but also provides comprehensive insights into the untapped potential of microbial biosynthetic capabilities, establishing genome mining as a foundational pillar of modern natural product research.

## 6. Cheminformatics Infrastructure

Cheminformatics has evolved into an indispensable component of the NP discovery pipeline, providing the robust analytical tools required to navigate complex chemical spaces [67]. Structural similarity algorithms identify relationships between novel isolates and known bioactive scaffolds, accelerating dereplication and analogue identification [68]. Quantitative Structure–Activity Relationship (QSAR) modeling correlates molecular descriptors with biological endpoints, guiding rational optimization [69]. To gain atomistic resolution of these interactions, researchers utilize molecular docking and molecular dynamics (MD) simulations [70]. These computational techniques allow for the detailed exploration of ligand-protein binding affinities and conformational stability, effectively streamlining lead prioritization and reducing the resource expenditure associated with traditional in vitro screening.

The integration of AI and ML has significantly enhanced the predictive accuracy of lead discovery and optimization workflows [71]. These technologies excel at processing large, heterogeneous datasets to identify non-linear bioactivity patterns, often surpassing traditional statistical methods in both speed and precision. ML algorithms are now routinely deployed to predict ADMET profiles, aiding in the early identification of promising drug candidates while mitigating potential clinical failures [27]. Deep learning architectures, in particular, have demonstrated success in uncovering complex bioactivity relationships, as evidenced by the identification of novel antimicrobial and oncology agents from vast natural product libraries. The shift toward data-driven NP discovery has been enabled by a new generation of computational resources. Table 3 summarizes key bioinformatics and cheminformatics tools that form the backbone of the modern digital workflow—from genome mining and metabolomic profiling to AI-powered prediction and chemical data curation. Together, they represent the essential technological toolkit for translating biological and chemical data into actionable drug leads.

**Table 3:** Essential Bioinformatics and Cheminformatics Resources in Modern NP Discovery. This table outlines core computational tools and platforms that have accelerated the “digital renaissance” in NP research. By transitioning from manual, low-throughput methods to automated, predictive workflows, these resources enable researchers to systematically decode, analyze, and harness nature’s chemical complexity at scale.

Resource Type	Tool/Database	Primary Function	Target Data Type	Strengths and Limitations	Reference
Genome Mining	antiSMASH	Automated BGC identification & annotation	Genomic/Metagenomic	**Strengths:** Gold standard for automated annotation. **Limitations:** High potential for over-prediction; requires manual curation for cryptic clusters.	[60]
Metabolomics	GNPS	Global Natural Products Social Molecular Networking	Mass Spectrometry (MS/MS)	**Strengths:** Unmatched for visualizing chemical families. **Limitations:** Performance is heavily dependent on the quality of user-submitted spectra.	[72]
Clustering	BiG-SCAPE	Sequence similarity networking of BGCs	Genomic/BGCs	**Strengths:** Excellent for comparative genomics. **Limitations:** Sequence-similarity thresholds can be arbitrary and miss divergent clusters.	[66]
Deep Learning	DeepBGC	AI-based BGC discovery using RNNs	Genomic sequences	**Strengths:** Capable of finding novel BGC classes. **Limitations:** “Black-box” nature makes it difficult to interpret the biological basis of predictions.	[73]
Cheminformatics	DataWarrior	Chemical data visualization and SAR analysis	Molecular scaffolds	**Strengths:** Highly intuitive GUI for visual analytics. **Limitations:** Scalability issues when handling massive multi-omics datasets.	[74]
Database	COCONUT	Collection of Open Natural Products	Chemical structures	**Strengths:** Comprehensive, open-access repositories. **Limitations:** Metadata consistency and annotation depth vary across entries.	[75]

## 7. Applications in Drug Discovery and Development

The power of contemporary informatics extends far beyond initial discovery, fundamentally reshaping the entire therapeutic lifecycle of NP scaffolds [76]. By mining and integrating multi-dimensional datasets, researchers can now repurpose established NPs and accelerate the engineering of next-generation leads with unprecedented efficiency [77]. This data-driven re-evaluation allows for the discovery of “off-target” interactions that can be therapeutically exploited, effectively reducing the temporal and financial costs of development. A notable example is the polyene antifungal Amphotericin B. While its ability to target cancer stem cell phenotypes has been experimentally demonstrated, subsequent MD simulations have provided the computational rationale, revealing how AmB distinguishes between the sterol-rich membranes of tumor cells versus healthy mammalian cells based on van der Waals interaction energies [78].

Aspirin (acetylsalicylic acid), derived from the NP salicin, continues to see its therapeutic profile expand through data analytics, with established roles in cardiology and emerging evidence supporting its chemopreventive potential in oncology [79]. Central to this roadmap is the computational prioritization of metabolites, a process that filters large-scale -omics data to rank ‘hits’ based on their structural novelty and predicted biological relevance. This workflow typically utilizes a ‘Novelty Index’ (comparing BGCs to the MIBiG database) and ‘Bioactivity Scores’ (derived from deep learning models) to bypass known chemistry and focus resources exclusively on the most promising dark-matter scaffolds.

The practical utility of these computational roadmaps is best seen in how they bridge the gap between raw genomic data and actual chemical discovery. This was exemplified by the discovery of Pyrrolomycin K and L [80], which were identified not through traditional screening, but through a computational prioritization pipeline that integrated *de novo* genome sequencing with molecular networking. Researchers used these tools to prioritize and isolate novel antimicrobials, like those found in termite-associated microbes by using ecological insights to guide the mining of biosynthetic gene clusters [80]. This synergy extends to extreme environments, where metabologenomics-driven strategies, specifically pairing 2D-NMR-metabolomics with genome mining have successfully activated silent pathways to isolate compounds like nocardimicins from psychrophilic strains [81]. Furthermore, the persistent bottleneck of dereplication is being dismantled by machine learning frameworks trained on in silico fragmentation spectra. These models can classify bioactivity directly from LC-MS/MS data with over 93% accuracy, bypassing the need for experimental reference spectra and significantly accelerating the prioritization of novel bioactive scaffolds [82].

In the context of lead optimization, informatics frameworks have become essential for compressing the timeline from “hit” to “lead” [17]. The synthesis of high-throughput screening (HTS) technologies with machine learning algorithms allows for the rapid evaluation of thousands of metabolites against diverse biological targets [83]. Furthermore, in silico modeling of ADMET properties ensures that only compounds with viable pharmacokinetic profiles progress through the pipeline, thereby mitigating the high attrition rates traditionally associated with NP research [52]. Recent workflows often integrate molecular docking with machine learning-driven structural modifications to identify derivatives with enhanced bioavailability and reduced systemic toxicity [53].

## 8. Challenges and Limitations

The informatics-driven transformation of NP discovery has created unprecedented opportunities for therapeutic development. However, this progress is constrained by significant technical limitations and unresolved ethical considerations that must be addressed to ensure both scientific rigor and equitable practice.

A fundamental technical challenge lies in the quality and accessibility of the underlying data. Public repositories of natural product information frequently suffer from inconsistent annotation standards and incomplete metadata regarding biosynthetic origins, extraction methodologies, and comprehensive bioactivity profiles [84]. This missing data problem often leads to inconsistencies in predictive models, limiting the reliability of machine learning-driven lead identification. Furthermore, while big data platforms have streamlined early-stage discovery, the computational scalability required to integrate and analyze multi-source omics datasets in real-time remains a significant resource constraint for many research institutions [85].

Beyond data accessibility, the inherent bias in training datasets poses a significant risk to the predictive reliability of AI models [86]. Current natural product databases are heavily skewed toward well-characterized taxonomic groups, such as *Streptomyces* [87] and certain filamentous fungi [88], and established chemical classes like polyketides and non-ribosomal peptides. This taxonomic and chemical space bias can lead to AI models that excel at identifying analogues of known compounds but struggle to accurately predict the bioactivity or biosynthetic boundaries of truly novel ‘dark matter’ scaffolds. Such systemic bias necessitates the development of transfer learning and few-shot learning techniques that can generalize from small, high-quality datasets to broader, underexplored chemical spaces.

Apart from technical constraints, ethical and legal considerations have become increasingly prominent, particularly concerning biodiversity conservation and biopiracy [18]. The exploitation of genetic resources from biodiverse regions necessitates rigorous adherence to equitable benefit-sharing frameworks, often governed by the Nagoya Protocol [89,90]. Navigating these international legalities is essential for fostering sustainable collaborations between academia, industry, and indigenous communities. Additionally, the management of Intellectual Property Rights (IPR) for NPs and their semi-synthetic derivatives remains a complex domain, as patenting often intersects with traditional ecological knowledge [91]. The development of NPs derived from or inspired by traditional medicines raises complex questions about attribution, compensation, and the protection of cultural heritage alongside scientific innovation.

Addressing these interconnected challenges requires coordinated efforts across multiple domains. Technically, the field must establish standardized reporting frameworks and promote the adoption of FAIR (Findable, Accessible, Interoperable, Reusable) data principles to enhance data quality and interoperability. Ethically, developing transparent frameworks for benefit-sharing and intellectual property that respect both scientific innovation and traditional knowledge will be essential for sustainable research partnerships. Only through such integrated approaches can the field fully realize the potential of data-driven natural product discovery while ensuring its practice remains scientifically robust and ethically sound.

## 9. Future Directions and Opportunities

The future of NP research is increasingly defined by the adoption of systems biology frameworks [92]. By situating natural products within the context of entire biological networks, researchers can map the intricate regulation of biosynthetic pathways and their holistic interactions with human targets [93]. Furthermore, synthetic biology is poised to play a transformative role, enabling the rational redesign of biosynthetic gene clusters (BGCs) to produce “unnatural” NPs with optimized pharmacological profiles [94,95]. This not only addresses supply chain challenges but also paves the way for designing “unnatural” NPs with enhanced therapeutic properties.

Advancements in AI-driven automation are expected to establish a new benchmark for efficiency, where robotic platforms perform real-time hypothesis testing, from in silico prediction to automated microfluidic screening [96,97]. This shift toward precision medicine offers a unique opportunity to leverage patient-specific genomic data, allowing NP scaffolds to be tailored to align with individual metabolic profiles [98,99]. Finally, the development of hybrid drug candidates, which merge NP scaffolds with synthetic moieties, represents a potent strategy for overcoming multidrug resistance and improving therapeutic efficacy [100]. Together, these advancements promise to not only revitalize NP research but also redefine its role in modern drug discovery and development. The sustainability of these innovations will rely on the implementation of specialized bioinformatics platforms, utilizing scalable workflows such as Nextflow and Galaxy to democratize access to complex genomic analyses for the broader scientific community.

## 10. Conclusions

The integration of big data analytics with NP discovery marks a definitive paradigm shift in pharmaceutical science. This digital renaissance has systematically deconstructed historical bottlenecks, replacing serendipitous bioprospecting with predictive, hypothesis-driven workflows powered by genome mining, multi-omics integration, and machine intelligence. By transforming NPs from scarce chemical curiosities into digitally accessible, design-ready scaffolds, the field has secured its critical role in the future of therapeutic innovation.

Sustaining this progress demands a concerted commitment to interdisciplinary convergence and infrastructural equity. The most pressing challenges—from data standardization and computational scalability to ethical sourcing and equitable benefit-sharing—cannot be solved within traditional disciplinary silos. They require collaborative frameworks that unite bioinformaticians, synthetic biologists, chemists, ethnobotanists, and legal scholars. Furthermore, investment in open, harmonized computational platforms and reproducible analytical workflows will be crucial for democratizing access and ensuring that the benefits of data-driven discovery are globally shared.

Ultimately, this synergy between nature’s chemical legacy and computational foresight offers a powerful blueprint for addressing complex human diseases. As we refine these technologies and navigate their ethical implications, NPs will continue to serve as an indispensable foundation for developing the next generation of precise, sustainable, and accessible medicines.

## Data Availability

Data sharing is not applicable to this article as no datasets were generated or analyzed during the current study.

## References

[1] Beutler J.A. (2009). Natural Products as a Foundation for Drug Discovery. Curr. Protoc. Pharmacol..

[2] Fleming A. (1944). The Discovery Of Penicillin. Br. Med. Bull..

[3] Wani M.C., Taylor H.L., Wall M.E., Coggon P., McPhail A.T. (1971). Plant antitumor agents. VI. The isolation and structure of taxol, a novel antileukemic and antitumor agent from Taxus brevifolia. J. Am. Chem. Soc..

[4] Atanasov A.G., Zotchev S.B., Dirsch V.M., Supuran C.T., International Natural Product Sciences Taskforce (2021). Natural products in drug discovery: Advances and opportunities. Nat. Rev. Drug Discov..

[5] Zhang Q.W., Lin L.G., Ye W.C. (2018). Techniques for extraction and isolation of natural products: A comprehensive review. Chin. Med..

[6] Atanasov A.G., Waltenberger B., Pferschy-Wenzig E.M., Linder T., Wawrosch C., Uhrin P., Temml V., Wang L., Schwaiger S., Heiss E.H. (2015). Discovery and resupply of pharmacologically active plant-derived natural products: A review. Biotechnol. Adv..

[7] Beutler J.A. (2019). Natural Products as a Foundation for Drug Discovery. Curr. Protoc. Pharmacol..

[8] Xie T., Song S., Li S., Ouyang L., Xia L., Huang J. (2015). Review of natural product databases. Cell Prolif..

[9] Winter J.M., Behnken S., Hertweck C. (2011). Genomics-inspired discovery of natural products. Curr. Opin. Chem. Biol..

[10] Ayon N.J. (2023). High-Throughput Screening of Natural Product and Synthetic Molecule Libraries for Antibacterial Drug Discovery. Metabolites.

[11] Demarque D.P., Dusi R.G., de Sousa F.D.M., Grossi S.M., Silverio M.R.S., Lopes N.P., Espindola L.S. (2020). Mass spectrometry-based metabolomics approach in the isolation of bioactive natural products. Sci. Rep..

[12] Caesar L.K., Montaser R., Keller N.P., Kelleher N.L. (2021). Metabolomics and genomics in natural products research: Complementary tools for targeting new chemical entities. Nat. Prod. Rep..

[13] Paul D., Sanap G., Shenoy S., Kalyane D., Kalia K., Tekade R.K. (2021). Artificial intelligence in drug discovery and development. Drug Discov. Today.

[14] Harvey A.L., Edrada-Ebel R., Quinn R.J. (2015). The re-emergence of natural products for drug discovery in the genomics era. Nat. Rev. Drug Discov..

[15] Newman D.J., Cragg G.M. (2020). Natural Products as Sources of New Drugs over the Nearly Four Decades from 01/1981 to 09/2019. J. Nat. Prod..

[16] Brown D.G., Bostrom J. (2016). Analysis of Past and Present Synthetic Methodologies on Medicinal Chemistry: Where Have All the New Reactions Gone?. J. Med. Chem..

[17] Thomford N.E., Senthebane D.A., Rowe A., Munro D., Seele P., Maroyi A., Dzobo K. (2018). Natural Products for Drug Discovery in the 21st Century: Innovations for Novel Drug Discovery. Int. J. Mol. Sci..

[18] Imran Y., Wijekoon N., Gonawala L., Chiang Y.C., De Silva K.R.D. (2021). Biopiracy: Abolish Corporate Hijacking of Indigenous Medicinal Entities. Sci. World J..

[19] Hong J. (2011). Role of natural product diversity in chemical biology. Curr. Opin. Chem. Biol..

[20] Dias D.A., Urban S., Roessner U. (2012). A historical overview of natural products in drug discovery. Metabolites.

[21] Huigens R.W., Morrison K.C., Hicklin R.W., Flood T.A., Richter M.F., Hergenrother P.J. (2013). A ring-distortion strategy to construct stereochemically complex and structurally diverse compounds from natural products. Nat. Chem..

[22] Ertl P., Schuhmann T. (2019). A Systematic Cheminformatics Analysis of Functional Groups Occurring in Natural Products. J. Nat. Prod..

[23] Bitchagno G.T.M., Nchiozem-Ngnitedem V.A., Melchert D., Fobofou S.A. (2022). Demystifying racemic natural products in the homochiral world. Nat. Rev. Chem..

[24] Galloway W.R., Isidro-Llobet A., Spring D.R. (2010). Diversity-oriented synthesis as a tool for the discovery of novel biologically active small molecules. Nat. Commun..

[25] Nasim N., Sandeep I.S., Mohanty S. (2022). Plant-derived natural products for drug discovery: Current approaches and prospects. Nucleus.

[26] Wall M.E., Wani M.C. (1995). Camptothecin and taxol: Discovery to clinic--thirteenth Bruce F. Cain Memorial Award Lecture. Cancer Res..

[27] LaPlante K.L., Woodmansee S. (2009). Activities of daptomycin and vancomycin alone and in combination with rifampin and gentamicin against biofilm-forming methicillin-resistant Staphylococcus aureus isolates in an experimental model of endocarditis. Antimicrob. Agents Chemother..

[28] Menon V.P., Sudheer A.R. (2007). Antioxidant and anti-inflammatory properties of curcumin. Adv. Exp. Med. Biol..

[29] Ahmad M., Ahmed Z., Yang X., Can M. (2023). Natural Resources Depletion, Financial Risk, and Human Well-Being: What is the Role of Green Innovation and Economic Globalization?. Soc. Indic. Res..

[30] Fleming A. (1929). On the antibacterial action of cultures of a penicillium, with special reference to their use in the isolation of B. influenzae. Br. J. Exp. Pathol..

[31] Holton R.A., Kim H.B., Somoza C., Liang F., Biediger R.J., Boatman P.D., Shindo M., Smith C.C., Kim S. (1994). First total synthesis of taxol. 2. Completion of the C and D rings. J. Am. Chem. Soc..

[32] Witthauer K. (1899). Aspirin, ein neues Salicylpräparat. Die Heilkunde.

[33] McCormick M.H., McGuire J.M., Pittenger G., Pittenger R., Stark W. (1955). Vancomycin, a new antibiotic. I. Chemical and biologic properties. Antibiot. Annu..

[34] Pelletier P.J., Caventou J.-B. (1820). Recherches Chimiques sur les Quinquinas.

[35] Endo A. (1985). Compactin (ML-236B) and related compounds as potential cholesterol-lowering agents that inhibit HMG-CoA reductase. J. Med. Chem..

[36] Lomovskaya N., Otten S.L., Doi-Katayama Y., Fonstein L., Liu X.C., Takatsu T., Inventi-Solari A., Filippini S., Torti F., Colombo A.L. (1999). Doxorubicin overproduction in Streptomyces peucetius: Cloning and characterization of the dnrU ketoreductase and dnrV genes and the doxA cytochrome P-450 hydroxylase gene. J. Bacteriol..

[37] Borel J.F., Feurer C., Gubler H.U., Stahelin H. (1976). Biological effects of cyclosporin A: A new antilymphocytic agent. Agents Actions.

[38] You-You T., Mu-Yun N., Yu-Rong Z., Lan-Na L., Shu-Lian C., Mu-Qun Z., Xiu-Zhen W., Zheng J., Xiao-Tian L. (1982). Studies on the constituents of Artemisia annua Part II. Planta Med..

[39] McGuire J.M., Bunch R.L., Anderson R.C., Boaz H.E., Flynn E.H., Powell H.M., Smith J.W. (1952). Ilotycin, a new antibiotic. Schweiz. Med. Wochenschr..

[40] Oppenheimer A. (1937). Turmeric (curcumin) in biliary diseases. Lancet.

[41] Sensi P. (1983). History of the development of rifampin. Rev. Infect. Dis..

[42] van Santen J.A., Jacob G., Singh A.L., Aniebok V., Balunas M.J., Bunsko D., Neto F.C., Castano-Espriu L., Chang C., Clark T.N. (2019). The Natural Products Atlas: An Open Access Knowledge Base for Microbial Natural Products Discovery. ACS Cent. Sci..

[43] Banerjee P., Erehman J., Gohlke B.O., Wilhelm T., Preissner R., Dunkel M. (2015). Super Natural II--a database of natural products. Nucleic Acids Res..

[44] Gaulton A., Bellis L.J., Bento A.P., Chambers J., Davies M., Hersey A., Light Y., McGlinchey S., Michalovich D., Al-Lazikani B. (2012). ChEMBL: A large-scale bioactivity database for drug discovery. Nucleic Acids Res..

[45] Scherlach K., Hertweck C. (2021). Mining and unearthing hidden biosynthetic potential. Nat. Commun..

[46] Qiu S., Cai Y., Yao H., Lin C., Xie Y., Tang S., Zhang A. (2023). Small molecule metabolites: Discovery of biomarkers and therapeutic targets. Signal Transduct. Target. Ther..

[47] Draisma A., Loureiro C., Louwen N.L.L., Kautsar S.A., Navarro-Munoz J.C., Doering D.T., Mouncey N.J., Medema M.H. (2026). BiG-SCAPE 2.0 and BiG-SLiCE 2.0: Scalable, accurate and interactive sequence clustering of metabolic gene clusters. Nat. Commun..

[48] Zdouc M.M., Blin K., Louwen N.L.L., Navarro J., Loureiro C., Bader C.D., Bailey C.B., Barra L., Booth T.J., Bozhuyuk K.A.J. (2025). MIBiG 4.0: Advancing biosynthetic gene cluster curation through global collaboration. Nucleic Acids Res..

[49] Poynton E.F., van Santen J.A., Pin M., Contreras M.M., McMann E., Parra J., Showalter B., Zaroubi L., Duncan K.R., Linington R.G. (2025). The Natural Products Atlas 3.0: Extending the database of microbially derived natural products. Nucleic Acids Res..

[50] Saldivar-Gonzalez F.I., Aldas-Bulos V.D., Medina-Franco J.L., Plisson F. (2022). Natural product drug discovery in the artificial intelligence era. Chem. Sci..

[51] Sahayasheela V.J., Lankadasari M.B., Dan V.M., Dastager S.G., Pandian G.N., Sugiyama H. (2022). Artificial intelligence in microbial natural product drug discovery: Current and emerging role. Nat. Prod. Rep..

[52] Wu F., Zhou Y., Li L., Shen X., Chen G., Wang X., Liang X., Tan M., Huang Z. (2020). Computational Approaches in Preclinical Studies on Drug Discovery and Development. Front. Chem..

[53] Singh S., Gupta H., Sharma P., Sahi S. (2024). Advances in Artificial Intelligence (AI)-assisted approaches in drug screening. Artif. Intell. Chem..

[54] Yang K., Han X. (2016). Lipidomics: Techniques, Applications, and Outcomes Related to Biomedical Sciences. Trends Biochem. Sci..

[55] Cho H., Qu Y., Liu C., Tang B., Lyu R., Lin B.M., Roach J., Azcarate-Peril M.A., Aguiar Ribeiro A., Love M.I. (2023). Comprehensive evaluation of methods for differential expression analysis of metatranscriptomics data. Brief. Bioinform..

[56] Mwalili S.M., Lesaffre E., Declerck D. (2008). The zero-inflated negative binomial regression model with correction for misclassification: An example in caries research. Stat. Methods Med. Res..

[57] Huang L., Song M., Shen H., Hong H., Gong P., Deng H.W., Zhang C. (2023). Deep Learning Methods for Omics Data Imputation. Biology.

[58] Meesil W., Muangpat P., Sitthisak S., Rattanarojpong T., Chantratita N., Machado R.A.R., Shi Y.M., Bode H.B., Vitta A., Thanwisai A. (2023). Genome mining reveals novel biosynthetic gene clusters in entomopathogenic bacteria. Sci. Rep..

[59] Kountz D.J., Balskus E.P. (2021). Leveraging Microbial Genomes and Genomic Context for Chemical Discovery. Acc. Chem. Res..

[60] Medema M.H., Blin K., Cimermancic P., de Jager V., Zakrzewski P., Fischbach M.A., Weber T., Takano E., Breitling R. (2011). antiSMASH: Rapid identification, annotation and analysis of secondary metabolite biosynthesis gene clusters in bacterial and fungal genome sequences. Nucleic Acids Res..

[61] Blin K., Shaw S., Vader L., Szenei J., Reitz Z.L., Augustijn H.E., Cediel-Becerra J.D.D., de Crecy-Lagard V., Koetsier R.A., Williams S.E. (2025). antiSMASH 8.0: Extended gene cluster detection capabilities and analyses of chemistry, enzymology, and regulation. Nucleic Acids Res..

[62] Zhong Z., He B., Li J., Li Y.X. (2020). Challenges and advances in genome mining of ribosomally synthesized and post-translationally modified peptides (RiPPs). Synth. Syst. Biotechnol..

[63] Albarano L., Esposito R., Ruocco N., Costantini M. (2020). Genome Mining as New Challenge in Natural Products Discovery. Mar. Drugs.

[64] Belknap K.C., Park C.J., Barth B.M., Andam C.P. (2020). Genome mining of biosynthetic and chemotherapeutic gene clusters in Streptomyces bacteria. Sci. Rep..

[65] Moellering R.C. (2011). Discovering new antimicrobial agents. Int. J. Antimicrob. Agents.

[66] Navarro-Munoz J.C., Selem-Mojica N., Mullowney M.W., Kautsar S.A., Tryon J.H., Parkinson E.I., De Los Santos E.L.C., Yeong M., Cruz-Morales P., Abubucker S. (2020). A computational framework to explore large-scale biosynthetic diversity. Nat. Chem. Biol..

[67] Chen Y., Kirchmair J. (2020). Cheminformatics in Natural Product-based Drug Discovery. Mol. Inform..

[68] Schenone M., Dancik V., Wagner B.K., Clemons P.A. (2013). Target identification and mechanism of action in chemical biology and drug discovery. Nat. Chem. Biol..

[69] Kwon S., Bae H., Jo J., Yoon S. (2019). Comprehensive ensemble in QSAR prediction for drug discovery. BMC Bioinform..

[70] Agu P.C., Afiukwa C.A., Orji O.U., Ezeh E.M., Ofoke I.H., Ogbu C.O., Ugwuja E.I., Aja P.M. (2023). Molecular docking as a tool for the discovery of molecular targets of nutraceuticals in diseases management. Sci. Rep..

[71] Gupta R.R. (2022). Application of Artificial Intelligence and Machine Learning in Drug Discovery. Methods Mol. Biol..

[72] Wang M., Carver J.J., Phelan V.V., Sanchez L.M., Garg N., Peng Y., Nguyen D.D., Watrous J., Kapono C.A., Luzzatto-Knaan T. (2016). Sharing and community curation of mass spectrometry data with Global Natural Products Social Molecular Networking. Nat. Biotechnol..

[73] Hannigan G.D., Prihoda D., Palicka A., Soukup J., Klempir O., Rampula L., Durcak J., Wurst M., Kotowski J., Chang D. (2019). A deep learning genome-mining strategy for biosynthetic gene cluster prediction. Nucleic Acids Res..

[74] Sander T., Freyss J., von Korff M., Rufener C. (2015). DataWarrior: An open-source program for chemistry aware data visualization and analysis. J. Chem. Inf. Model..

[75] Sorokina M., Merseburger P., Rajan K., Yirik M.A., Steinbeck C. (2021). COCONUT online: Collection of Open Natural Products database. J. Cheminform..

[76] Wright G.D. (2014). Something old, something new: Revisiting natural products in antibiotic drug discovery. Can. J. Microbiol..

[77] Glicksberg B.S., Li L., Chen R., Dudley J., Chen B. (2019). Leveraging Big Data to Transform Drug Discovery. Methods Mol. Biol..

[78] Boukari K., Balme S., Janot J.M., Picaud F. (2016). Towards New Insights in the Sterol/Amphotericin Nanochannels Formation: A Molecular Dynamic Simulation Study. J. Membr. Biol..

[79] Montinari M.R., Minelli S., De Caterina R. (2019). The first 3500 years of aspirin history from its roots—A concise summary. Vascul. Pharmacol..

[80] Lin M., de Kruijff M., Poulsen M., Beemelmanns C. (2025). Genome-Mining Based Discovery of Pyrrolomycin K and L from the Termite-Associated *Micromonospora* sp. RB23. J. Nat. Prod..

[81] Xu S., Wang N., Meng Q., Ma W., Li H. (2025). Metabologenomics-Driven Discovery of Nocardimicins from a *Psychrophilic nocardia* sp. Strain. J. Nat. Prod..

[82] Brittin N.J., Anderson J.M., Braun D.R., Rajski S.R., Currie C.R., Bugni T.S. (2025). Machine Learning-Based Bioactivity Classification of Natural Products Using LC-MS/MS Metabolomics. J. Nat. Prod..

[83] Szymanski P., Markowicz M., Mikiciuk-Olasik E. (2012). Adaptation of high-throughput screening in drug discovery-toxicological screening tests. Int. J. Mol. Sci..

[84] Sorokina M., Steinbeck C. (2020). Review on natural products databases: Where to find data in 2020. J. Cheminform..

[85] Yang A., Troup M., Ho J.W.K. (2017). Scalability and Validation of Big Data Bioinformatics Software. Comput. Struct. Biotechnol. J..

[86] Norori N., Hu Q., Aellen F.M., Faraci F.D., Tzovara A. (2021). Addressing bias in big data and AI for health care: A call for open science. Patterns.

[87] Feng Y., Qaseem A., Moumbock A.F.A., Pan S., Kirchner P.A., Simoben C.V., Malange Y.I., Babiaka S.B., Gao M., Gunther S. (2025). StreptomeDB 4.0: A comprehensive database of streptomycetes natural products enriched with protein interactions and interactive spectral visualization. Nucleic Acids Res..

[88] Xu Y., Yang D., Guo Z., Wu N., Shi T., Zhang C. (2025). Advances and Potential of Aspergillus niger in Industrial Biomanufacturing. J. Agric. Food Chem..

[89] Efferth T., Banerjee M., Paul N.W., Abdelfatah S., Arend J., Elhassan G., Hamdoun S., Hamm R., Hong C., Kadioglu O. (2016). Biopiracy of natural products and good bioprospecting practice. Phytomedicine.

[90] Ortiz A.M.D., Outhwaite C.L., Dalin C., Newbold T. (2021). A review of the interactions between biodiversity, agriculture, climate change, and international trade: Research and policy priorities. One Earth.

[91] Kartal M. (2007). Intellectual property protection in the natural product drug discovery, traditional herbal medicine and herbal medicinal products. Phytother. Res..

[92] Najmi A., Javed S.A., Al Bratty M., Alhazmi H.A. (2022). Modern Approaches in the Discovery and Development of Plant-Based Natural Products and Their Analogues as Potential Therapeutic Agents. Molecules.

[93] Altaf-Ul-Amin M., Afendi F.M., Kiboi S.K., Kanaya S. (2014). Systems biology in the context of big data and networks. Biomed Res. Int..

[94] Zhang M.M., Wang Y., Ang E.L., Zhao H. (2016). Engineering microbial hosts for production of bacterial natural products. Nat. Prod. Rep..

[95] Roychowdhury R., Das S.P., Gupta A., Parihar P., Chandrasekhar K., Sarker U., Kumar A., Ramrao D.P., Sudhakar C. (2023). Multi-Omics Pipeline and Omics-Integration Approach to Decipher Plant’s Abiotic Stress Tolerance Responses. Genes.

[96] Zhu H. (2020). Big Data and Artificial Intelligence Modeling for Drug Discovery. Annu. Rev. Pharmacol. Toxicol..

[97] Gaudencio S.P., Bayram E., Lukic Bilela L., Cueto M., Diaz-Marrero A.R., Haznedaroglu B.Z., Jimenez C., Mandalakis M., Pereira F., Reyes F. (2023). Advanced Methods for Natural Products Discovery: Bioactivity Screening, Dereplication, Metabolomics Profiling, Genomic Sequencing, Databases and Informatic Tools, and Structure Elucidation. Mar. Drugs.

[98] Mushtaq S., Abbasi B.H., Uzair B., Abbasi R. (2018). Natural products as reservoirs of novel therapeutic agents. EXCLI J..

[99] Atanasov A.G., Yeung A.W.K., Banach M. (2018). Natural products for targeted therapy in precision medicine. Biotechnol. Adv..

[100] Miethke M., Pieroni M., Weber T., Bronstrup M., Hammann P., Halby L., Arimondo P.B., Glaser P., Aigle B., Bode H.B. (2021). Towards the sustainable discovery and development of new antibiotics. Nat. Rev. Chem..

